# The Development of Dorsal Nasal Cyst Formation after Rhinoplasty and Its Reconstruction with Conchal Cartilage

**DOI:** 10.1155/2014/928715

**Published:** 2014-05-06

**Authors:** Tolgar Lütfi Kumral, Yavuz Uyar, Güven Yıldırım, Güler Berkiten, Yavuz Atar, Enes Ataç

**Affiliations:** Department of Otorhinolaryngology-Head and Neck Surgery, Okmeydanı Training and Research Hospital, Darülaceze Caddesi No. 25, Okmeydanı, Şişli, Istanbul, Turkey

## Abstract

The dorsal nasal cyst formation is a rare and late complication of rhinoplasty. It has been rarely reported in the literature and it is usually mucous cysts. Migration and planting to the subcutaneous space during the surgical procedure has been recognized as the formation mechanism. This case report has presented 42-year-old male patient with a destructing dorsal nasal mucous cyst that developed 10 years after the rhinoplasty operation. There was no complication in the primary rhinoplasty and the patient was satisfied with his appearance. There was a swelling of the nasal dorsum over the past year and surgical excision of the cyst was performed. During the surgery, the defect was reconstructed with conchal cartilage. There was no recurrence during follow-up.

## 1. Introduction

The dorsal nasal cyst formation is a rare and late complication of rhinoplasty. Current literature contains only a few case reports about this complication and these are mostly mucous cysts [1].

Different tissues such as bone, cartilage, and soft tissue can be affected during surgical procedures. Also, it is difficult to predict the outcome of surgery due to the dynamic nature of the healing process.

The complications of rhinoplasty can be classified as early and late complications. Early complications are generally related to intraoperative sterility and surgical technique [2]. Late complications are related to resorption of cartilage [3].

The assumed mechanism of dorsal nasal cyst formation is thought to be the migration and seeding of surgical debridement to the subcutaneous tissue during surgery [3]. In a few cases, the foreign inclusion body had been reported inside of cyst formation due to placing graft material [4, 5].

In our study, we aimed to discuss a case about development of cystic formation in the nasal dorsum ten years after rhinoplasty.

## 2. Case Presentation

**Figure 1:**
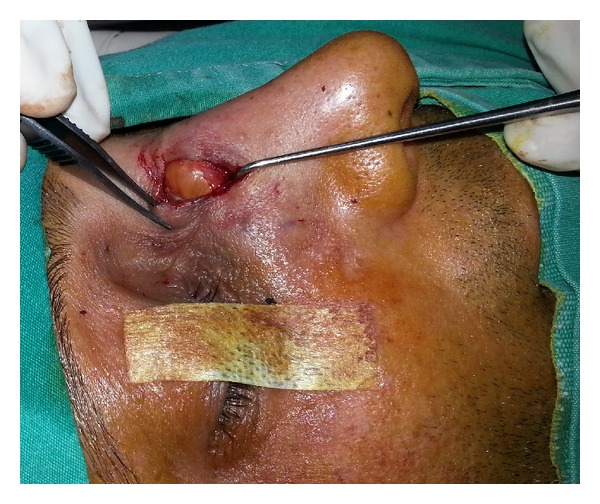
Intraoperative lateral view of the cystic formation on the nasal dorsum.

A forty-two-year-old man presented to us with right nasal radix mass which was increasing in size within the last year. The patient had undergone rhinoplasty surgery and there were no complications after the procedure. The physicial examination has revealed a 2 cm painless mass with smooth borders, cystic in nature, and it has caused erosion in the nasal dorsum through the nasal bone (Figure 1). Nasal tip was normal, without any deviation, and no intranasal mass was noted.

**Figure 2:**
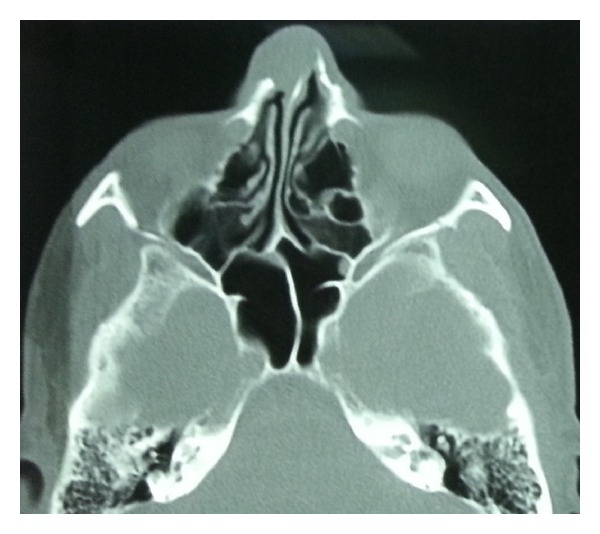
Heterogeneous density cystic mass destroying the nasal bone in the axial tomography of the temporal bone.

**Figure 3:**
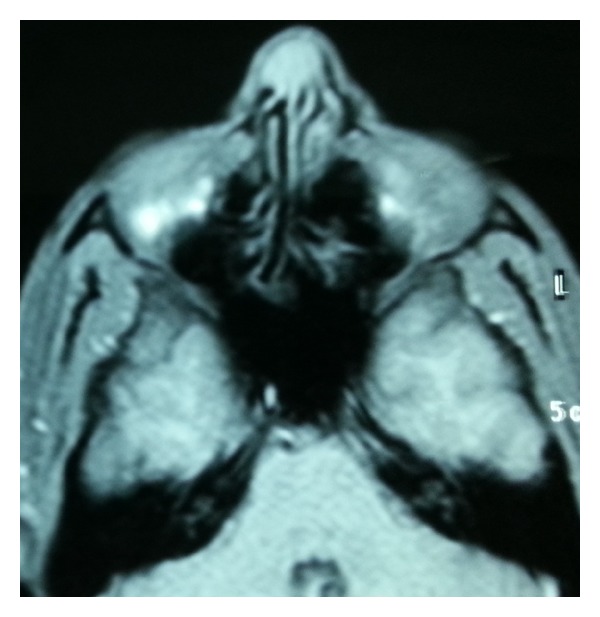
Cystic mass in T2-weighted cranial MRI on the right side of the nasal dorsum.

Computer tomography showed a heterogeneous mass which caused erosion in nasal dorsum through the nasal bone. Cranial MRI (T2A sequence) showed a cystic mass which was not spreaded into any intracranial structures (Figures 2-3).

**Figure 4:**
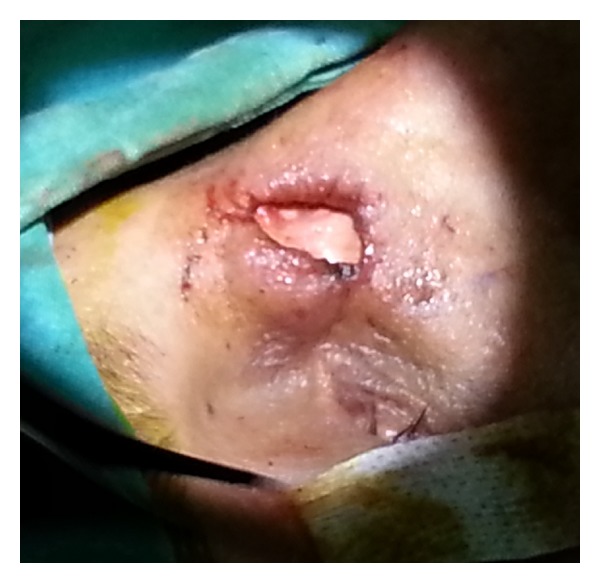
Reconstruction of the dorsal nasal defect with conchal cartilage after excision of the cyst.

The patient was informed about the risks and benefits of the procedure and informed consent was obtained from patient. After that, the operation was done under general anesthesia. Incision was made from sterile skin to the depth of the mass during operation. The mass, which eroded nasal dorsum and involed the nasal cavity, was dissected from surrounding tissues and entirely excised. A graft taken from conchal cartilage was laid over the defect according to its shape and then it was sutured to the surrounding tissue (Figure 4).

The excised cystic tissue was well-circumscribed and contained brown, serous fluid. Final pathology result has reported that the tissue has an inflammatory polypoid formation with respiratory tract epithelium. No complications were observed during follow-up period. Also, no defects were observed cosmetically. The patient did not complain of any scar formation at his first year of check-up.

## 3. Discussion

Rhinoplasty is a very common surgery nowadays. The most important expectations of patients and surgeons from this surgery are pleasing from cosmetic results and no recurrence of any deformity over the years. However, many complications may develop even after years from rhinoplasty.

Tissue edema, periorbital ecchymosis, infection, skin necrosis, and numbness can be seen in the early period after rhinoplasty surgery. Early period deformities can occur mostly due to mistakes in surgical technique. The most important cause of late period revison surgery is postoperative deformities which composes of approximately 5–15% of all causes [2].

The development of mucous cyst formation is a rare complication in the late period. Generally, it can be observed in the nasal dorsum along the nasal osteotomy line. The migration and seeding into the subcutaneous space was accepted by authors as the mechanism of injury. It has been believed that this usually occurs after a traumatic surgical technique. Karapantzos et al. [6] have reported a case that mucous cyst occurred on the maxillary osteotomy line in the paranasal region.

All cyst cases have supported the seeding theory till now and then they were characterized as being successfully treated with surgery without any recurrence.

Interestingly, one case report has reported a cyst that developed from mandibular respiratory epithelium in patient underwent genioplasty and rhinoplasty surgery. In this case, it has shown that using osteocartilaginous material for chin augmentation caused the implantation [7].

The most important way to avoid these complications is preparing the graft by separating it from the epithelium as much as possible and avoiding unnecessary trauma during the osteotomy.

After rhinoplasty, developed cysts are usually mucoid in nature and arise from respiratory epithelium. However, a case has been reported about development of epidermoid inclusion cysts in posttraumatic patients after rhinoplasty.

The most important point in the treatment of developed dorsal nasal cyst is complete excision of cyst and reconstruction of nasal dorsum [8]. In this case, our patient did not have problem related to nasal type and lesion was located far above from nasal type. Therefore, reconstruction was made by an external incision. This surgical procedure can be done by transcolumellar incision with open septorhinoplasty surgery. Autografts should be considered as a revision material; the grafts that will create foreign body reaction should be avoided. In the literature, many cases have reported foreign body reaction related to graft materials [9].

The most important issue for used graft materials on the nasal dorsum is causing the irregularities that can be seen from the outside. Therefore, graft material should be carefully selected and placed properly.

Septal, costal, or conchal cartilage can be used for reconstruction. In this case, our patient previously had septoplasty; thus he did not have sufficient cartilage remaining; therefore, we used the conchal cartilage for reconstruction surgery. Nasal dorsum was supported with autogen chonchal graft. By this way, we have created a natural cosmetic appearance. The conchal cartilage was slightly sloped and erosional area was also sloped. So it was made in a better way.

The dorsal nasal cyst formation is a rare and late complication of rhinoplasty. The most important thing to avoid this complication is to be sure from cleaning all of the epithelium from the prepared graft and avoiding any unnecessary trauma. Complications seen after rhinoplasty can be reduced by increased surgical experience.
